
JOURNAL INFORMATION
==============================
NLM Title Abbreviation: Crohns Colitis 360
Journal ID: Crohns Colitis 360
NLM Journal ID: 101752188
Journal ID: crohnscolitis360

Crohn's & Colitis 360

EISSN: 2631-827X

ARTICLE INFORMATION
==============================
PMCID: PMC7184458
PMID: 38626280
DOI: 10.1093/crocol/otaa029
Article ID: otaa029
Article version: 1
Subjects: Editorial Commentary

IBD in the Time of COVID19

Moss Alan MD Alan.Moss@bmc.org
Regueiro Miguel MD
1 Boston Medical Center , UNITED STATES
2 Chair, Department of Gastroenterology, Hepatology, Nutrition , UNITED STATES
Corresponding Email: Alan.Moss@bmc.org
Electronic publication date: 2020 Apr 16
Electronic Location ID: otaa029
Received 2020 Apr 6
Copyright: © 2020 Crohn’s & Colitis Foundation. Published by Oxford University Press on behalf of Crohn’s & Colitis Foundation.
Copyright year: 2020
License: This is an Open Access article distributed under the terms of the Creative Commons Attribution License (http://creativecommons.org/licenses/by/4.0/), which permits unrestricted reuse, distribution, and reproduction in any medium, provided the original work is properly cited.
License URL: https://creativecommons.org/licenses/by/4.0/

Comment in: The Risk of SARS-CoV-2 in Immunosuppressed IBD Patients 2 2 16 4 2020 otaa026 Crohn's & Colitis 360 10.1093/crocol/otaa026 PMC7184434 32377636
Comment in: The Risk of SARS-CoV-2 in Immunosuppressed IBD Patients 2 2 16 4 2020 otaa026 Crohn's & Colitis 360 10.1093/crocol/otaa026 PMC7184434 32377636
article-lifecycle: PAP
edited-state: accepted-manuscript

==============================
**IBD in the Time of COVID19**

Patients and healthcare teams are facing unprecedented stresses as a result of the current severe acute respiratory syndrome coronavirus 2 (SARS-CoV-2) global pandemic. We are always conscious of the risk of infections in patients with Crohn's disease and Ulcerative Colitis, but the global burden of COVID19 has raised many questions for us as clinicians. To provide some data and reference points to address these questions, we are pleased to publish a 'hot topic' review by Higgins, Danese, Ng and Rao in this issue of CC360. They share experience from Asia and Italy, and ongoing collaborative efforts worldwide. As data about COVID19 emerges on a daily basis, our goal is to provide a medium for rapid dissemination of peer-reviewed work on its relevance to IBD. The Open access digital format of CC360 allows universal access to this information without any paywalls. At a time when many of us feel overwhelmed with the scope of this SARS-CoV-2 pandemic, we will continue to focus on informing our community with the most relevant information for managing IBD.

"Knowledge itself is power" Sir Francis Bacon, *Mediationes Sacrae* (1597)

Alan Moss, MD

Miguel Regueiro, MD

Accepted Manuscript

© 2020 Crohn's & Colitis Foundation. Published by Oxford University Press on behalf of Crohn's & Colitis Foundation.
This is an Open Access article distributed under the terms of the Creative Commons Attribution License (http://creativecommons.org/licenses/by/4.0/), which permits unrestricted reuse, distribution, and reproduction in any medium, provided the original work is properly cited.
